# Bridging the first-aid knowledge gap: a cross-sectional study of medical scope students in Syria

**DOI:** 10.1017/S1463423624000033

**Published:** 2024-02-08

**Authors:** Jamal Ataya, Jawdat Ataya, Ziad Aljarad

**Affiliations:** 1 Faculty of Medicine, University of Aleppo, Aleppo, Syria; 2 Dental Medicine, Damascus University, Damascus, Syria; 3 Medical Education, Syrian Virtual University, Damascus, Syria; 4 Internal Medicine Department, Faculty of Medicine, University of Aleppo, Aleppo, Syria

**Keywords:** first-aid, medical students, Syria, Syrian medical students

## Abstract

**Introduction and Objective:**

Sufficient knowledge required to deal with emergencies at the accident site may not be found in most medical students due to the lack of effective first-aid training in most medical education curricula. This study aims to assess and evaluate medical students’ knowledge level in providing first-aid care, especially first-year students.

**Methods:**

An electronic questionnaire was distributed via social media to 1,855 medical students in October 2020. The knowledge level was assessed based on scores obtained for each clinical scenario requiring first aid and classified as good, intermediate, or weak. Statistical analysis was performed using SPSS software.

**Results:**

The study found that academic year and specialization significantly influence medical students’ first-aid knowledge level. However, demographic factors such as gender, university, marital status, housing status, work status, financial condition, and previous first-aid training did not show any significant effect.

**Conclusion:**

The level of knowledge among Syrian medical students in providing first-aid care is somewhat limited. Therefore, first-aid courses should be made more accessible to these students, and their effectiveness should be ensured and maintained through frequent updates. Moreover, more attention should be placed on publicizing first-aid knowledge to make life-saving procedures attainable to anyone, anytime and anywhere.

## Introduction

First aid provides immediate care to people injured or struck by a sudden injury until support from an expert or the injured person recovers. The primary purposes of first aid are to preserve life, relieve pain as much as possible, prevent further complications, and stabilize the patients (Pek, 2017) (Van de Velde *et al.*, 2009). Furthermore, anyone can be exposed to injury in various situations, whether at work or at home. Providing immediate and accurate first-aid care at the accident site is crucial (Joseph *et al.*, 2014) (Abbas, Bukhari and Ahmad, 2011). Based on the gathered information, we found that assessments of first-aid knowledge among medical students and those involved in the medical sector have been commonly discussed in the Middle East, but similar studies have not been conducted in Syria, particularly during the crisis. Furthermore, many medical students did not understand first-aid principles and practices, especially in their early academic years (Midani *et al.*, 2019). In addition, also exists a lack of adequate published research or reports discussing this topic (Tan *et al.*, 2006). Therefore, is essential to publicize first-aid knowledge among all age groups and social workers, particularly those in the medical sector, because they belong to the first-line professionals on life defense within critical situations. Also, they should handle the situation with caution and without panic (Hafen, Mistovich and Karren, 2011). This study is priceless because it deals with a critical issue in Syria for the first time, especially after long suffering from wars that left behind an urgent need to learn the principles of first aid.

## Methods

### Study Design

We conducted a descriptive study to assess first-aid knowledge among medical students in Syrian universities and colleges for all academic years, and the survey lasted for ten days between 10/10/2020 till 20/10/2020. The study was conducted through an electronic evaluation questionnaire due to difficulties reaching all Syrian governorates.

### Participants & Data Collection

All participants were current undergraduate medical and health science students at public and private universities in Syria. Participants were enrolled from different medical fields, including medicine, dentistry, pharmacy, nursing, health sciences, and medical technology. An anonymous online questionnaire was designed using Google Forms and distributed in Arabic in groups that included large numbers of students in medicine and health fields via online medical platforms in all Syrian governorates to reach the required sample due to the coronavirus pandemic. The study’s aims were explained to the participants, who were informed that their participation was voluntary and guaranteed anonymity. The participants were also informed that this research results would be published. All missing data have been filtered out. The study was approved by the Ethical Committee of the Faculty of Medicine at Aleppo University with a serial number (1112/3598) and complied with the principles of the Helsinki Declaration (‘World Medical Association Declaration of Helsinki: ethical principles for medical research involving human subjects’, 2013).

### Questionnaire

The questionnaire was designed by trauma and first-aid specialists according to the latest British first-aid guidelines (see Supplementary file). Then, a pilot study was conducted on 100 students, and the questionnaire was revised by deleting and modifying some options according to the preliminary statistical study. The questions were selected based on British guidelines to ensure a comprehensive evaluation of all the theoretical knowledge essentials of first aid among students concerned with the medical field. This selection was informed by similar studies and standard first-aid research. Also, the survey was tested for reliability by using Cronbach’s alpha test. Internal consistency of (0.809) was reported.

The questionnaire consists of 29 questions directed to Syrian university and college students concerned with the medical sector. The questions were divided into two groups.

The first group contained 15 questions divided into two parts. Part one included personal information such as gender, age, academic year, and marital status. The second part included whether the student had taken any first-aid courses as shown in Additional file (1).

The second group contained 14 questions assessing all major first-aid knowledge aspects as shown in Additional file (2).

### Statistical Analysis

The data were automatically exported from Google forms to Excel, and analyses were performed using Statistical Package for Social Sciences software package (SPSS Inc., Chicago, IL, USA) version 23. One-way analysis of variance (ANOVA) was performed to consider the overall differences in mean knowledge scores. The chi-square test was used to find out the association of demographic variables with the level of knowledge regarding first aid, and *P* < 0.05 was taken as a statistically significant association. The approximate population of undergraduate medical and health science students at public and private Syrian universities was between 20000 to 21000 in the 2020 census, with a 95% confidence level, margin of error of 0.05 and a confidence interval (CI) of 1.5; the required sample size was 377.

## Results

Initially, about 2013 questionnaires were collected. Afterwards, the sample was inspected and processed, and all unfinished questionnaires have been deleted. About 1855 questionnaires were obtained to form the final sample. Students of various medical specialities were classified according to multiple measures, including academic specialization, gender, academic years, university, marital status, accommodation, work status, and financial level. 93.9% of participants were from Syrian public medical colleges and 6.1% from Syrian Private medical colleges. Among those, 50.5% were studying medicine, 19.5% were studying Pharmacy, 11.9% were studying dentistry, 9.8 were studying nursing, 7.6% were studying medical technology and 0.7 were studying health sciences. Female participants counted for 65.7% of the sample versus 34.3% as males. Other sample details and characteristics are shown in Additional file 1. The questions that evaluate the level of knowledge of the principles of first aid shows in Additional file (2).

We have allocated one point (1) to each question from the second questionnaire group for each true answer and zero points (0) to the wrong answers. The questions are an integrated unit so that a gap within one answer eliminates part of the participant’s final knowledge score. Thus, the total score is anywhere from 0 if all answers were wrong to 14 if all answers were correct. Three levels of knowledge were adopted, namely weak level from (0) to (4) were 454 (24.4%), intermediate level from (5) to (9) were 1205 (65%), and good level from (10) to (14) were 196 (10.6%). Total scores are shown in Table 1. After calculating the level of students’ knowledge of the first aid, we found the mean, median and std. Deviations were 6.36, 6.00 and 2.577, respectively. When comparing the level of knowledge of students of medical colleges, including medicine, pharmacy, dentistry, and others, with gender, marital status, public or private university, financial level, working status, and accommodation, it was found that there was an insignificant difference when we statistically used Chi-Square Test for each of them.

**Table 1.** Total scores for the level of knowledge of the principles of first aid

		Level of knowledge					
		Weak		Intermediate		Good	
		Count	Column *n*%	Count	Column *n*%	Count	Column *n*%
University specialization:	Faculty of Medicine	237	52.3%	607	50.3%	94	48.0%
	Faculty of Dentistry	60	13.2%	143	11.9%	17	8.7%
	Faculty of Pharmacy	72	15.9%	240	19.9%	50	25.5%
	School of Nursing	53	11.7%	112	9.3%	16	8.2%
	Faculty of Health Sciences	2	0.4%	8	0.7%	3	1.5%
	Medical Technology Institute	29	6.4%	96	8.0%	16	8.2%

On the other hand, we conducted a chi-square test to assess the relationship between students’ academic majors and their current academic year and level of knowledge in first-aid management. The test revealed a statistically significant association (χ^2^ (65, *n* = 1855) = 99.846, *P* = 0.023*). Specifically, we found a significant difference between their academic majors and the academic year they were currently studying with the level of knowledge in first-aid management.

In these specialized fields, distinct patterns emerged in the distribution of students achieving high scores (above 10) and low scores (below 5). For example, within the medical school, 938 students were examined, with 48% demonstrating a strong understanding of the subject matter, while 52.3% obtained scores below 5. Among the 220 dentistry students, 8.7% achieved high scores, whereas 13.2% received low scores. Among the 362 pharmacy students, 25.5% obtained high scores, whereas 15.9% had low scores. Similarly, in a nursing program with 181 students, 8.2% displayed strong performance, whereas 11.7% exhibited weaker performance. These findings highlight the diverse academic outcomes within these specialized fields of study. Significantly, within the Health Science specialization, 1.5% of the 13 students achieved high scores, while 0.4% obtained low scores. It is imperative to note that the limited sample size in this category may have impacted the outcomes. In contrast, among 181 medical technology students, there was an 8.2% success rate in achieving a high level of performance, while 6.4% recorded scores below 5.

The analysis of the relationship between academic year and student performance revealed a highly significant association (χ^2^ (65, *n* = 1855) = 136.717, *P* = 0.000**). Notably, the results displayed a discernible pattern, with first-year students (278) exhibiting a relatively low success rate of only 3.3% in achieving good scores (above 10). However, this rate progressively increased as students advanced through their academic years. By the sixth year, the total sample size was 67, and they achieved the highest success rate, reaching 6.7%. This observed trend implies an enhancement in student performance as they progress through their academic journey.

Finally, when studying the relationship of their knowledge of the principles of first aid with their previous first-aid courses, the *P*-value was equal to 0.073, which is very close to 0.05, meaning that it did not affect much.

## Discussion

In this study and a similar study at the University of Mosul and Lucknow, very few students demonstrated good knowledge of first-aid principles, whether they had received prior training in first-aid procedures or not (Makhlef, 2014)(Gore *et al.*, 2017). Likewise, a Peruvian study among medical students reported that 60.4% of the participants showed poor first-aid knowledge, although 52.5% had received prior training on how to act in an emergency. Further, a Dutch study reported that 81% of junior doctors had poor knowledge of first-aid principles (Mejia *et al.*, 2011). On the contrary, were a study in a Saudi university showed positive results, where more than half of the participants had good knowledge about the principles of first aid (AlQahtani *et al.*, 2020). Thus, we conclude that taking courses alone is insufficient to apply the knowledge in real-life situations. Therefore, it is advisable to perform frequent knowledge assessments throughout the courses to obtain the best possible result. It is also encouraged to undergo follow-up sessions every 6–12 months to ensure that the population in general, and medical students in particular, remain well-informed and updated about the latest practices followed in first-aid procedures (Arshi *et al.*, 2006).

We also note that most medical students obtained average scores on their first-aid knowledge assessment and that only a tiny group scored above average. Furthermore, the largest proportion of clinical-year (senior) students scored well on first-aid knowledge assessment. This indicates the urgent need to focus more on first aid among pre-clinical students (Mejia *et al.*, 2011). In this study, 35.9% of students were correctly aware of the steps of cardiopulmonary resuscitation (CPR) as part of their overall first-aid administration, which was a very high percentage compared to the results of a study conducted in Salem, Tamil Nadu, where it was reported to be 17.1%. Also, in the Dutch study, only 6% of students knew and performed CPR correctly (Chandrasekaran, Kumar and Bhat, 2010). Nevertheless, except that similarly to our own, two Karachi-based studies reported that 32.2% and 38.8% of participants knew how to perform CPR correctly (Tan *et al.*, 2006). Moreover, the test results were similar to some studies and different from others, as we have shown previously, depending on the different causes leading to this result and the different living conditions in the countries. Properly administering first aid for burns was recognized by 57% of students compared to 23.2% in an Irish study, showing that our students are thoroughly informed about the concept of burns. On the other hand, the latter study reported that 30.4% of medical students had good knowledge of first-aid management in cases of accidental ingestion of toxins compared to 21.9% in our study (Abbas, Bukhari and Ahmad, 2011).

As for applying ambulance-aid principles to fractures, external bleeding, and cases of trauma in general, many students demonstrated good knowledge, indicating that our curricula focus relatively more on these topics. In contrast, regarding the priority of examination when viewing Injured, a low percentage of students answered correctly, comprising about 32.2%. Moreover, based on the results obtained Additional file 2, we find that most of the students did not provide correct answers on several topics, such as ankle sprain and trauma, which comprised 26% of the lack of knowledge of trauma management compared to fractures, whose rate was only about 8.1%.

We discovered a noteworthy distinction in first-aid knowledge among medical students from different specializations, with pharmacy students demonstrating the highest level of proficiency. The reason for the difference might be that pharmacy students are trained more comprehensively in first aid than other medical students. In the United States, pharmacy students are mandated to take a course in first aid and cardiopulmonary resuscitation (CPR), while other medical students are not required to take any formal training in first aid. One possible reason for the discrepancy in first-aid knowledge between pharmacy and other medical students is that pharmacy students tend to have more hands-on experience with first aid. For instance, pharmacy students may provide first aid to customers in pharmacies, whereas other medical students may have limited opportunities to practice such skills (*Departments of Labor and Health, education, and welfare and Related Agencies Appropriations for Fiscal Year 1978 hearings before a subcommittee of the Committee on Appropriations, United States Senate, Ninety-fifth Congress, First Session, on H.R. 7555*, 1977) (Mertz *et al.*, 2021) (Alsharif *et al.*, 2023).

It is essential to acknowledge that our research was observational, which means we cannot confirm a direct causal connection between medical specialization and first-aid proficiency. Nonetheless, our discoveries imply that all medical students in Syria would benefit from receiving extensive first-aid training. This would guarantee that they are prepared to offer immediate assistance in any emergency scenario. Furthermore, other research has also indicated that comprehensive first-aid knowledge is crucial for medical students regardless of their field of specialization. For example, Al-Qahtani et al. determined that medical students with more proficient first-aid knowledge were more likely to intervene during a medical emergency (AlQahtani *et al.*, 2020). Moreover, Basuhail et al. discovered that first-aid training enhanced medical students’ self-assurance and capacity to manage medical crises (Basuhail *et al.*, 2022).

Increased knowledge among medical students would result in knowledgeable and confident individuals handling any critical situation they might encounter; this may be especially vital for a country in the face of any natural disasters because medical students will be the future cornerstone of facing any health risk in society may be exposed to. Another benefit of training students in first aid is that they can successfully provide first-aid training to their peers or other beneficiaries, as stated by 97.7% of medical students at Altintaş et al. (Khan *et al.*, 2010) (Altntas *et al.*, 2009) (Altintas *et al.*, 2005).

## Conclusion

In general, students in clinical years performed more reasonable than students in earlier years. Furthermore, this study emphasizes the need to provide training courses for medical students, particularly first-aid courses, so that trained students can perform effective first-aid procedures independently and spontaneously in real-life situations. We also note that there is currently no formal first-aid training in medical curricula for students in their early years. Therefore, there is an urgent need for first-aid training among medical and pre-clinical students. The study also identified areas where a deficiency of knowledge among students was prevalent, and therefore, these areas must be the main priority of future curriculum revisions. Finally, we note that more studies should be conducted to assess first-aid knowledge and other healthcare-related skills among medical students in Syria.

## Supporting information

Ataya et al. supplementary material 1 Ataya et al. supplementary material

Ataya et al. supplementary material 2 Ataya et al. supplementary material

## Data Availability

All data generated or analyzed during this study are included in this published article.

## Supplementary material

To view supplementary material for this article, please visit https://doi.org/10.1017/S1463423624000033.

## Limitation

1. The lack of dependable curricula and official information on first aid in Syria.
2. There is no trustworthy official funding.
