# Supplementary material for: Bridging the first-aid knowledge gap: a cross-sectional study of medical scope students in Syria
Source: Prim Health Care Res Dev. 2024 Feb 8;25:e8. doi: 10.1017/S1463423624000033 (PMC10894718; doi:10.1017/S1463423624000033)
Supplement: Ataya et al. supplementary material 1 — Ataya et al. supplementary material [file S1463423624000033sup001.docx]

| **Additional file 1:** The basic demographic information of the sample and whether the student took any previous first-aid courses | | | |
| --- | --- | --- | --- |
|  | | **Count** | **Column N %** |
| **Academic Specialization** | other | 0 | 0.0% |
|  | Faculty of Medicine | 938 | 50.5% |
|  | Faculty of Dentistry | 220 | 11.9% |
|  | Faculty of Pharmacy | 362 | 19.5% |
|  | School of Nursing | 181 | 9.8% |
|  | Faculty of Health Sciences | 13 | 0.7% |
|  | Medical Technology Institute | 141 | 7.6% |
| Gender | Male | 636 | 34.3% |
|  | Female | 1219 | 65.7% |
| Academic Years | First year | 278 | 15.0% |
|  | Second year | 745 | 40.2% |
|  | Third year | 389 | 21.0% |
|  | Fourth year | 256 | 13.8% |
|  | Fifth year | 120 | 6.5% |
|  | Sixth year | 67 | 3.6% |
| Are you still a university student or have you finished your university level (you graduated from university) | Yes (student) | 1855 | 100.0% |
|  | No (graduate) | 0 | 0.0% |
| The university you are currently studying at | Damascus University | 928 | 50.0% |
|  | Kalamoon Private University | 22 | 1.2% |
|  | Al-Andalus Private University | 12 | 0.6% |
|  | Al-Hawash Private | 15 | 0.8% |
|  | Ittihad Private University (IPU) | 8 | 0.4% |
|  | Arab International University (AIU) | 3 | 0.2% |
|  | Aleppo University | 290 | 15.6% |
|  | Al-Baath University | 173 | 9.3% |
|  | Tishreen University | 200 | 10.8% |
|  | Tartous University | 51 | 2.7% |
|  | Hama University | 99 | 5.3% |
|  | Al-Sham Private University | 21 | 1.1% |
|  | Syrian Private University (SPU) | 25 | 1.3% |
|  | International University for Science and Technology (IUST) | 8 | 0.4% |
| Original residence before going to university | Damascus | 561 | 30.2% |
|  | Al-Hasakah | 48 | 2.6% |
|  | Al-Raqqa | 9 | 0.5% |
|  | Al-Suwayda | 74 | 4.0% |
|  | Quneitra | 13 | 0.7% |
|  | Idlib | 30 | 1.6% |
|  | Damascus Countryside | 323 | 17.4% |
|  | Aleppo | 211 | 11.4% |
|  | Homs | 136 | 7.3% |
|  | Tartous | 110 | 5.9% |
|  | Lattakia | 130 | 7.0% |
|  | Hama | 117 | 6.3% |
|  | Daraa | 69 | 3.7% |
|  | Der Al-Zoor | 24 | 1.3% |
| Family status | Single | 1815 | 97.8% |
|  | Married | 33 | 1.8% |
|  | Widower/ widow | 3 | 0.2% |
|  | Divorced | 4 | 0.2% |
| Accommodation | With my friends | 139 | 7.5% |
|  | Alone | 55 | 3.0% |
|  | With my relatives | 68 | 3.7% |
|  | University accommodation | 279 | 15.0% |
|  | With my family | 1314 | 70.8% |
| Do you work during the study period | Yes | 289 | 15.6% |
|  | No | 1566 | 84.4% |
| What is your financial level in general | very good | 464 | 25.0% |
|  | good | 1286 | 69.3% |
|  | bad | 105 | 5.7% |
| Have you ever attended a first aid course? | Yes | 646 | 34.8% |
|  | No | 1209 | 65.2% |

| **Additional file 2:** The questions that evaluate the level of knowledge of the principles of first aid | | | |
| --- | --- | --- | --- |
|  | | **Count** | **Column N %** |
| What is the priority of the examination according to the principles of first aid when viewing an injured person in general | I do not no | 166 | 8.9% |
|  | Breath - Response - Airways - Circulatory System | 436 | 23.5% |
|  | Airways - Breath - Response - Circulatory System | 363 | 19.6% |
|  | Response - Airways - Breath - Circulatory | 597 | 32.2% |
|  | Circulatory System - Breath - Airways - Response | 293 | 15.8% |
| When a person suffers from complete obstruction of the airway, the following procedure should be performed | I do not no | 293 | 15.79% |
|  | stroking between shoulder blades | 341 |  |
|  | Heimlich maneuver | 1133 | 61.07% |
|  | Inciting the patient to vomit | 68 |  |
|  | Give the patient person a glass of water to open the Airways | 20 |  |
| External bleeding is managed according to the principles of first aid by | I do not no | 72 | 3.9% |
|  | Wait until the bleeding has stopped and then cover the wound with a specific cloth. | 45 | 2.4% |
|  | Sterilize the wound with the available sterilizers. | 196 | 10.6% |
|  | Apply manual pressure | 1542 | 83.1% |
| Nosebleeds are managed by | I do not no | 106 | 5.7% |
|  | Apply pressure to the cartilage section of the nose and head forward. | 1108 | 59.7% |
|  | Never press the nose and tilt the head back. | 91 | 4.9% |
|  | Apply pressure to the cartilaginous section of the nose and head backward. | 354 | 19.1% |
|  | Never press the nose and tilt the head forward. | 196 | 10.6% |
| How is the shock state treated? | I do not no | 360 | 19.4% |
|  | Reassure, then cover, then extend the patient, then lift the legs. | 167 | 9.0% |
|  | Reassure, then extend the patient, then lift the legs, then cover. | 608 | 32.7% |
|  | Extend the patient, then lift the legs, then reassure, then cover. | 218 | 11.8% |
|  | Extend the patient, then lift the legs, then cover then reassure. | 502 | 27.1% |
| We do not give the shock patient any food or drink even when requested. | I do not no | 609 | 32.8% |
|  | True | 905 | 48.8% |
|  | False | 341 | 18.4% |
| The main symptoms of a fracture | I do not no | 150 | 8.1% |
|  | absence of pain - functional disability - absence of deformation of the broken organ- swelling | 74 | 4.1% |
|  | Severe pain - functional disability - deformation of the broken organ - swelling | 1457 | 78.5% |
|  | Severe pain - functional disability- absence of deformation of the broken organ - absence of swelling | 114 | 6.1% |
|  | Moderate pain - functional disability - deformation of the broken organ - absence of swelling | 60 | 3.2% |
| An ankle sprain is treated by | I do not no | 512 | 27.6% |
|  | RICE procedure R:Rest I:Ice C:Comfortable E:Elevate | 468 | 25.2% |
|  | Massage the area with ointments | 57 | 3.1% |
|  | Fixing the area with a compressive strap | 767 | 41.3% |
|  | Wash the area with warm water | 51 | 2.7% |
| First and superficial burns are managed according to the principles of first aid through | I do not no | 115 | 6.2% |
|  | Wash the burning place with cold or running water - cover the burn after it has cooled | 1343 | 72.4% |
|  | Put toothpaste on the burn site to cool it down | 77 | 4.2% |
|  | Cover the burn site only | 18 | 1.0% |
|  | Apply ointment to the burn and cover the place of the burn | 302 | 16.2% |
| All material stuck to the place of burning in third-degree burns is removed according to the principles of first aid | I do not no | 529 | 28.5% |
|  | True | 557 | 30.0% |
|  | False | 769 | 41.5% |
| When the patient is poisoned with the drug as a result of an overdose, the patient is induced to vomit according to the principles of first aid | I do not no | 258 | 13.9% |
|  | True | 1191 | 64.2% |
|  | False | 406 | 21.9% |
| The patient is given nitroglycerin four times every five minutes | I do not no | 1105 | 59.6% |
|  | True | 88 | 4.7% |
|  | False | 662 | 35.7% |
| The CPR cycle is | I do not no | 764 | 41.2% |
|  | 30 pressures /2 breaths per minute | 666 | 35.9% |
|  | 15 pressures / 2 breaths per minute | 226 | 12.2% |
|  | 30 pressures / 4 breaths per minute | 95 | 5.1% |
|  | 15 pressures / 4 breaths per minute | 104 | 5.6% |
| A patient with hyperglycemia is treated with | I do not no | 224 | 12.1% |
|  | Calling an ambulance and monitoring vital signs. | 232 | 12.5% |
|  | Give him a small amount of salt | 1245 | 67.1% |
|  | Lift the patient feet and calm down the patient | 75 | 4.0% |
|  | Give the patient an aspirin | 79 | 4.3% |
