# Supplementary material for: Bridging the first-aid knowledge gap: a cross-sectional study of medical scope students in Syria
Source: Prim Health Care Res Dev. 2024 Feb 8;25:e8. doi: 10.1017/S1463423624000033 (PMC10894718; doi:10.1017/S1463423624000033)
Supplement: Ataya et al. supplementary material 2 — Ataya et al. supplementary material [file S1463423624000033sup002.docx]

**Validated Questionnaire for Assessing First Aid Knowledge.**

**What is the priority of the examination according to the principles of first aid when viewing an injured person in general:**

**Note:** (Breath) refers to the inhale and exhale - (Airways) tract means the airways leading to the lungs - (Response) means the degree of awareness.

- Breath - Response - Airways - Circulatory System.
- Airways - Breath - Response - Circulatory System.
- **Response - Airways - Breath – Circulatory.**
- Circulatory System - Breath - Airways – Response.
- I do not no.

**When a person suffers from complete obstruction of the airway, the following procedure should be performed:**

- Stroking between shoulder blades.
- **Heimlich maneuver.**
- Inciting the patient to vomit.
- Give the patient person a glass of water to open the Airways.
- I do not no.

**External bleeding is managed according to the principles of first aid by:**

- Wait until the bleeding has stopped and then cover the wound with a specific cloth.
- Sterilize the wound with the available sterilizers.
- **Apply manual pressure.**
- I do not no.

**Nosebleeds are managed by:**

- **Apply pressure to the cartilage section of the nose and head forward.**
- Never press the nose and tilt the head back.
- Apply pressure to the cartilaginous section of the nose and head backward.
- Never press the nose and tilt the head forward.
- I do not no.

**How is the shock state treated?**

- Reassure, then cover, then extend the patient, then lift the legs.
- Reassure, then extend the patient, then lift the legs, then cover.
- Extend the patient, then lift the legs, then reassure, then cover.
- **Extend the patient, then lift the legs, then cover then reassure.**
- I do not no.

**We do not give the shock patient any food or drink even when requested:**

- **True.**
- False.
- I do not no.

**The main symptoms of a fracture:**

- absence of pain - functional disability - absence of deformation of the broken organ- swelling.
- **Severe pain - functional disability - deformation of the broken organ – swelling.**
- Severe pain - functional disability- absence of deformation of the broken organ - absence of swelling.
- Moderate pain - functional disability - deformation of the broken organ - absence of swelling.
- I do not no.

**An ankle sprain is treated by:**

- **RICE procedure, R:Rest I:Ice C:Comfortable E:Elevate.**
- Massage the area with ointments.
- Fixing the area with a compressive strap.
- Wash the area with warm water.
- I do not no.

**First and superficial burns are managed according to the principles of first aid through:**

- **Wash the burning place with cold or running water - cover the burn after it has cooled.**
- Put toothpaste on the burn site to cool it down.
- Cover the burn site only.
- Apply ointment to the burn and cover the place of the burn.
- I do not no.

**All material stuck to the place of burning in third-degree burns is removed according to the principles of first aid:**

- True.
- **False.**
- I do not no.

**When the patient is poisoned with the drug as a result of an overdose, the patient is induced to vomit according to the principles of first aid:**

- True.
- **False.**
- I do not no.

**The patient is given nitroglycerin four times every five minutes:**

- True.
- **False.**
- I do not no.

**The CPR cycle is:**

- **30 pressures /2 breaths per minute.**
- 15 pressures / 2 breaths per minute.
- 30 pressures / 4 breaths per minute.
- 15 pressures / 4 breaths per minute.
- I do not no.

**A patient with hyperglycemia is treated with:**

- **Calling an ambulance and monitoring vital signs – breathing, pulse and level of response.**
- Give him a small amount of salt.
- Lift the patient feet and calm down the patient
- Give the patient an aspirin.
- I do not no.

**Scoring:** Each correct answer is awarded 1 point, with a maximum possible score of 14 points. The scores can be categorized as follows: weak level of knowledge (0-4 points), intermediate level of knowledge (5-9 points), and good level of knowledge (10-14 points). The reliability of the survey was tested using the Cronbach’s alpha test, which reported an internal consistency of 0.809.
